# *Penicillium digitatum* as a Model Fungus for Detecting Antifungal Activity of Botanicals: An Evaluation on Vietnamese Medicinal Plant Extracts

**DOI:** 10.3390/jof8090956

**Published:** 2022-09-13

**Authors:** Hanh My Tran, Diep Hong Le, Van-Anh Thi Nguyen, Tao Xuan Vu, Nguyen Thi Kim Thanh, Do Hoang Giang, Nguyen Tien Dat, Hai The Pham, Marc Muller, Huy Quang Nguyen, Van-Tuan Tran

**Affiliations:** 1 Faculty of Biology, University of Science, Vietnam National University, Hanoi (VNU), 334 Nguyen Trai, Thanh Xuan, Hanoi 100000, Vietnam; 2 Laboratory for Organogenesis and Regeneration, Department of Life Sciences, GIGA-I3, University of Liège, 4000 Liège, Belgium; 3 Center for Experimental Biology, National Center for Technological Progress, Ministry of Science and Technology, C6 Thanh Xuan Bac, Thanh Xuan, Hanoi 100000, Vietnam; 4 Center for Research and Technology Transfer, Vietnam Academy of Science and Technology, 18 Hoang Quoc Viet, Cau Giay, Hanoi 100000, Vietnam; 5 National Key Laboratory of Enzyme and Protein Technology, University of Science, Vietnam National University, Hanoi (VNU), 334 Nguyen Trai, Thanh Xuan, Hanoi 100000, Vietnam

**Keywords:** antifungal activity, berberine, *Mahonia bealei*, Vietnamese medicinal plants, *Penicillium digitatum*, fungal pathogens

## Abstract

Medicinal plants play important roles in traditional medicine, and numerous compounds among them have been recognized for their antimicrobial activity. However, little is known about the potential of Vietnamese medicinal plants for antifungal activity. In this study, we examined the antagonistic activity of twelve medicinal plant species collected in Northern Vietnam against *Penicillium digitatum*, *Aspergillus flavus*, *Aspergillus fumigatus*, and *Candida albicans*. The results showed that the antifungal activities of the crude extracts from *Mahonia bealei*, *Ficus semicordata*, and *Gnetum montanum* were clearly detected with the citrus postharvest pathogen *P. digitatum*. These extracts could fully inhibit the growth of *P. digitatum* on the agar medium, and on the infected citrus fruits at concentrations of 300–1000 µg/mL. Meanwhile, the other tested fungi were less sensitive to the antagonistic activity of the plant extracts. In particular, we found that the ethanolic extract of *M. bealei* displayed a broad-spectrum antifungal activity against all four pathogenic fungi. Analysis of this crude extract by enrichment coupled with high-performance liquid chromatography revealed that berberine and palmatine are major metabolites. Additional inspections indicated berberine as the key compound responsible for the antifungal activity of the *M. bealei* ethanolic extract. Our study provides a better understanding of the potential of Vietnamese medicinal plant resources for combating fungal pathogens. This work also highlights that the citrus pathogen *P. digitatum* can be employed as a model fungus for screening the antifungal activity of botanicals.

## 1. Introduction

Medicinal plants and their secondary metabolites have been widely exploited in treating diseases worldwide, especially in Asian countries [1,2,3]. Diverse bioactive compounds of three major groups—terpenoids, alkaloids, and phenolic compounds—are synthesized and accumulated in different parts of plants, including flowers, fruits, leaves, stems, and roots. These compounds can be employed as the ingredients of antibiotic drugs, antiobesity agents, anticancer and antimicrobial agents, etc. [4,5,6,7]. Fungal infection appears to be a big threat to public health and agricultural production. The use of antifungal agents for treating fungal infections has led to drug-resistant strains. The emergence of resistant pathogens urges the demand for developing new antifungal strategies [8,9,10]. A survey of the ethanolic and aqueous extracts prepared from twenty plant species used in Palestinian folk medicine determined that ten of the tested plant extracts were able to inhibit the growth of the opportunistic yeast *Candida albicans*, of which the ethanolic extract of *Micromeria nervosa* was the most active [11]. An investigation of fourteen medicinal plants collected in Mongolia revealed that the extracts of four plants, *Stellaria dichotoma*, *Scutellaria scordifolia*, *Aquilegia sibirica*, and *Hyoscyamus niger*, displayed antifungal activities against *Candida* species, dermatophytes, and *Malassezia furfur*, which cause cutaneous diseases [12]. Analysis of fourteen medicinal plants collected in Canada identified three plants, *Fragaria virginiana*, *Epilobium angustifolium*, and *Potentilla simplex*, as possessing strong antifungal activities against pathogenic yeasts and molds. In particular, *F. virginiana* showed activity against all tested fungal pathogens [13]. Screening of the antifungal activities of ten medicinal plants from Ghana, it was indicated that the crude extracts of three plants, *Portulaca quadrifida*, *Ageratum conyzoides*, and *Newbouldia lavis*, had remarkable antifungal activity against the opportunistic fungus *Aspergillus fumigatus*. Notably, the plant *P. quadrifida* was demonstrated for the first time to possess antagonistic activity against both of the human pathogens *C. albicans* and *A. fumigatus* [14]. An examination of fourteen Indian medicinal plants showed that methanolic extracts of *Solanum xanthocarpum* and *Datura metel* significantly inhibited the growth of *A. fumigatus*, *Aspergillus flavus*, and *Aspergillus niger* [15]. Analysis of the chemical composition of *Berberis vulgaris* revealed the extract contained alkaloids with berberine as the active constituent. Berberine exhibits broad-spectrum antifungal activity and can be isolated from many medicinal plants [16,17]. Additional studies have also confirmed medicinal plants as promising sources for the discovery of novel antifungal compounds [18].

Medicinal plants and their phytochemicals have also been exploited as biofungicides for plant disease control and fruit preservation [19,20,21,22]. Many important fungal phytopathogens, such as *Magnaporthe oryzae*, *A. flavus*, *Fusarium* species, *Colletotrichum* species, *Penicillium* species, etc., have been successfully treated with plant-derived fungicides [9,21,23]. One of the most severe postharvest pathogens is *Penicillium digitatum*, a causative agent of green mold disease in citrus fruits. This necrotrophic fungus infects citrus fruits through injuries and accounts for approximately 90% of total postharvest losses [24]. Control of *P. digitatum* and other fungal plant pathogens relies on synthetic fungicides such as imazalil, thiabendazole, iprodione, fludioxonil, pyrimethanil, and prochloraz. However, the application of these synthetic fungicides raises public concerns regarding their adverse effects on human health and the environment. Furthermore, the frequent use of chemical agents in plant protection has favored the development of fungicide-resistant fungal strains [19,25]. Compared to synthetic fungicides, botanical products are quickly degraded in nature, and, therefore, are preferred in organic agriculture to control fungal pathogens [26,27].

Vietnam has a vast diversity of tropical plants that have been used in ethnomedicine for thousands of years [28,29,30,31,32,33,34]. Several studies have been conducted to evaluate the potential of Vietnamese medicinal plants for antiproliferative activity [35], gout treatment [36], antimalarial and cytotoxic activities [37], antioxidant and antidiabetic effects [38], and antibacterial activity [39]. An online database for Vietnamese herbal species with information on bioactive metabolites has recently been established [40]. In this study, we inspected crude extracts from twelve Vietnamese medicinal plants against four common pathogenic fungi. We found that three plants, including *M. bealei*, *F. semicordata*, and *G. montanum*, possess antifungal activities, and the crude extracts from these plants could be used for the preservation of citrus fruits. Furthermore, we also indicated that the citrus postharvest pathogen *P. digitatum* appears to be a promising model fungus for screening the antifungal activity of plant extracts.

## 2. Materials and Methods

### 2.1. Plant Material Samples

Twelve plant species commonly used in Vietnamese traditional medicine were collected from five different provinces of Northern Vietnam, including Lao Cai, Ha Giang, Quang Ninh, Hoa Binh, and Tuyen Quang. The plant samples were processed in the laboratory and dried at 45 °C to a constant weight using a hot air oven (Memmert GmbH, Schwabach, Germany). Plant samples were mechanically ground to fine powders and subjected to extraction. Plant specimens were deposited for reference at the Biological Museum, Faculty of Biology, University of Science, Vietnam National University, Hanoi (Table 1).

### 2.2. Fungal Strains and Spore Preparation

Four strains of different fungal species, including *P. digitatum* PdVN1 [41], *A. flavus* NRRL 3357 [42], *A. fumigatus* VTCC 30015 from Vietnam Type Culture Collection (http://vtcc.imbt.vnu.edu.vn (accessed on 6 September 2022)), and *C. albicans* JCM 2070 from Japan Collection of Microorganisms (https://www.jcm.riken.jp (accessed on 6 September 2022)) were chosen for antifungal activity assays of the plant extracts. These fungal strains were preserved at −80 °C as spore (cell) suspensions in 20% glycerol.

Potato dextrose agar (PDA) was used to cultivate *P. digitatum*, *A. flavus*, *A. fumigatus*, and *C. albicans*. For fungal spore suspension, 50 µL of a spore suspension (10^6^ spores/mL) was spread on a PDA plate. The plate was incubated for 4–5 days at 25–28 °C (for *P. digitatum*) or 30 °C (for *A. flavus* and *A. fumigatus*). A 10 mL volume of sterile distilled water was added to the surface of the plate, and spores were separated from fungal mycelium using a sterilized glass spreader. Spores were then harvested and resuspended in sterile distilled water at a concentration of 10^6^ spores/mL. For the preparation of a cell suspension of *C. albicans*, the yeast was cultured on PDA at 30 °C for 2 days. Yeast cells were harvested and resuspended in sterile distilled water at a concentration of 10^6^ cells/mL.

### 2.3. Preparation of Plant Extracts

Dried plant powders were used for extraction with two different solvents, including ethanol and distilled water. Each dried powder sample was suspended in the solvent at a ratio of 1:10 (*w*/*v*) and kept in an ultrasonic incubator (S100H, Elma GmbH, Singen, Germany) at 45 °C for 30 min. The mixture was swirled at 200 rpm for 60 min at room temperature using an orbital incubator shaker (Gyromax™ 747 Incubator Shaker, Amerex Instruments, Concord, CA, USA). The supernatant was separated from the plant residues by centrifugation (Avanti J-E, Beckman Coulter, Brea, CA, USA) at 10,000 rpm for 20 min at 10 °C. The supernatant was then filtered through Whatman No. 1 filter paper (Whatman, Buckinghamshire, UK). The filtrate was entirely dried in a vacuum rotary evaporator (HS-2005S-N, Hahnshin, Gimpo-si, Korea) or lyophilized (UniFreeze FD-8, Daihan Scientific, Wonju, Korea), depending on whether the solvent was ethanol or water, respectively. The dried extracts were stored at 4 °C. The crude extracts were dissolved in dimethyl sulfoxide (DMSO) to obtain stock solutions of 100 mg/mL. These stock solutions were then diluted to different concentrations for further analysis.

### 2.4. In Vitro Antifungal Activity Assays

The agar well diffusion method was used for the rapid detection of antifungal activity [43] with adjustments. Crude extracts of twelve medicinal plants were screened for their antifungal activity. A 50 µL volume of fungal spore suspension (10^6^ spores/mL) was spread onto the surface of a PDA plate. Afterward, 6-mm-diameter wells were punched and filled with 50 µL of each plant extract. DMSO (10%) was used as negative control. The plates were preincubated at 4 °C for 4 h to allow diffusion of potential antifungal substances into the agar medium. Subsequently, the plates were incubated at 25–30 °C for 3–4 days. Diameters of inhibition zones formed by antifungal activity were measured. The fungal inhibition was calculated using Formula (1):

FI = D − d(1)

where FI represents fungal inhibition (mm), D is the diameter of the inhibition zone appearing on the agar plate (mm), and d is the diameter of the agar well (mm).

Determination of minimal inhibitory concentration (MIC) was performed using the microdilution method [43] with adjustments: the stock extract solutions (100 mg/mL) were added to PDA medium to achieve final concentrations of 5, 10, 20, 40, 50, 100, 200, 300, 400, 500, 600, 700, 800, 900, and 1000 µg/mL. DMSO (10%) was used for negative control instead of the plant extracts. Then, 5 µL volumes of spore suspensions or yeast cells (10^4^, 10^5^, and 10^6^ spores (cells)/mL) were spotted onto the PDA plates containing a given extract concentration. Plates were incubated at 25 °C for 3 days (*P. digitatum*), 30 °C for 2 days (*A. flavus*, *A. fumigatus*), or 37 °C for 1 day (*C. albicans*).

### 2.5. Antifungal Activity Assays of Plant Extracts against P. digitatum on Citrus Fruits

Orange fruits were purchased from a local supermarket. Fruits were washed with tap water, dipped into 70% ethanol for 2 min, rinsed twice with sterile water for 10 min, and air-dried in a sterile cabinet. Artificial wounds (1.5 cm long × 2 mm deep) were made on the orange peel using a sterile metal utensil. The wounded fruits were immersed for 10 min in a clean glass beaker containing a solution supplemented with the plant extract at the MIC values. The fruits were dried at room temperature in a sterile cabinet. In the following steps, 10 µL of the *P. digitatum* spore suspension (10^3^ spores/mL) was inoculated to the wounded oranges, and the fruits were kept separately in sterile transparent plastic boxes for 2–6 days at 25 °C. Oranges inoculated with sterile water were used as negative control (mock). The experiments were performed with three independent replicates, and 3 fruits were used for each replicate.

### 2.6. Enrichment and Isolation of Alkaloids from M. bealei Ethanolic Extract

The *M. bealei* ethanolic crude extract was resuspended in 1N HCl at a ratio of 1:20 (*w*/*v*) and successively fractionated three times in ethyl acetate at proportions of 1:1 (*v*/*v*). Next, the acidic phase was alkalinized to pH 8–9 with a 2M NaOH solution and extracted three times with two volumes of dichloromethane. The organic phase was concentrated to collect the alkaloid enrichment. Then, 5.0 g of the enrichment was subjected to a silica gel chromatography column (CC) with gradient mixtures of dichloromethane—methanol (50:1–1:1, *v*/*v*) to afford eleven subfractions (F1–F11). Fraction F8 (1205 mg) was further separated on a silica gel CC eluted with dichloromethane–ethyl acetate–methanol (5/1/1, *v*/*v*/*v*) to obtain compound 1 (855 mg). Fraction F9 (573 mg) was chromatographed on a silica gel column and eluted with dichloromethane–methanol (10/1, *v*/*v*) to yield compound 2 (128 mg). The isolated compounds were identified by high-performance liquid chromatography with diode-array detection (HPLC-DAD) analysis under the same conditions as the corresponding reference compounds.

### 2.7. Quantitation of Berberine and Palmatine

The berberine and palmatine in the total extract and the alkaloid enrichment of *M. bealei* were quantitated using the HPLC-DAD method. HPLC-grade solvents were purchased from Scharlau (Barcelona, Spain) and Merck Millipore (Darmstadt, Germany). HPLC-DAD data were generated using a Thermo Ultimate 3000 system consisting of a quaternary pump, an autosampler, a column oven, and a diode array detector. A Hypersil GOLD HPLC column (250 mm × 4.6 mm, 5 µm) was used at 40 °C, while 0.1% (*v*/*v*) acetic acid in water and acetonitrile (ACN, Concord, NC, USA) were set as solvent channel A and B, respectively. A flow rate of 1.0 mL per min was selected, with a linear gradient from 25% to 50% ACN over 20 min, followed by 5 min washing with 100% methanol and 5 min of the initial condition. The injection volume for each sample was 2 µL. The UV detector was set at 345 nm (for the simultaneous determination of berberine and palmatine). Calibration solutions for each standard compound were prepared in a concentration range of 10–200 µg/mL and analyzed under the same conditions as the samples. Berberine and palmatine were purchased as standard compounds from Sigma-Aldrich (St. Louis, MI, USA).

### 2.8. Statistical Analysis

All the experiments were conducted in triplicate, and the data are expressed as mean ± SD. Statistical significance was determined by analysis with GraphPad Prism 8 (GraphPad Software, San Diego, CA, USA) using one-way ANOVA, and statistical difference was considered when *p* < 0.05.

## 3. Results

### 3.1. Three Medicinal Plant Species Possess Antifungal Activity

In this study, we inspected twenty-four crude extracts of twelve medicinal plant species (Table 1) for antifungal activity against the citrus postharvest pathogen *P. digitatum*, the aflatoxin-producing mold *A. flavus*, the human pathogenic filamentous fungus *A. fumigatus*, and the human pathogenic yeast *C. albicans*. These plants were collected from five different provinces of Northern Vietnam, where there is a diverse distribution of many traditional medicinal plants (Supplementary Materials Figure S1). The collected plant materials were mechanically treated and extracted with water or ethanol to obtain crude extracts. The agar diffusion assays showed that nine plants, including *T. chantrieri*, *C. cyrtophyllum*, *C. asiaticum*, *M. barbatus*, *A. balansae*, *H. capitellata*, *S. scandens*, *C. aloifolium*, and *T. sinensis*, exhibited no antifungal activity. However, we found that three plant crude extracts could suppress the growth of at least one of the tested pathogenic fungi. Antifungal activities of *M. bealei* (KT09) and *G. montanum* (KT11) were only detected when the plant materials were extracted with ethanol, whereas *F. semicordata* (KT10) showed antagonistic activity only when extracted with water (Table 2).

The obtained results suggest that the antifungal activity present in these extracts is soluble in different solvents and, thus, is probably due to divergent compounds. Additionally, the antagonistic activity of the *F. semicordata* water extract was restricted to only *P. digitatum*, while the ethanolic extract from *G. montanum* displayed mild antifungal activity against *P. digitatum*, *A. flavus*, and *A. fumigatus*. The *M. bealei* ethanolic extract exhibited broad-spectrum antifungal activity against all four tested pathogenic fungi. Antifungal activities of this extract against *A. flavus*, *A. fumigatus*, and *C. albicans* were further confirmed using the microdilution method. With a supplementation of 800 µg/mL of the KT09 extract to the PDA medium, the growth of *A. flavus*, as well as *C. albicans*, with an inoculum of 5000 spores (or 5000 cells), was nearly completely inhibited. Notably, the KT09 ethanolic extract was highly efficient at inhibiting *A. fumigatus* growth, as observed in both assays. The addition of KT09 ethanolic extract to the PDA medium at the final concentration of 40 µg/mL resulted in the complete inhibition of the growth of *A. fumigatus* (Supplementary Materials Figure S2).

In summary, the results reveal that *P. digitatum* is the most sensitive species for detecting antifungal activity in three plant extracts, either as ethanolic or water extract, which could also antagonize some of the other fungi tested. Therefore, we suggest that *P. digitatum* can be exploited as a model fungus for screening the antifungal activity of plant extracts from natural resources.

### 3.2. Evaluating Antifungal Activity of Three Plant Extracts against P. digitatum

**Figure 1:**
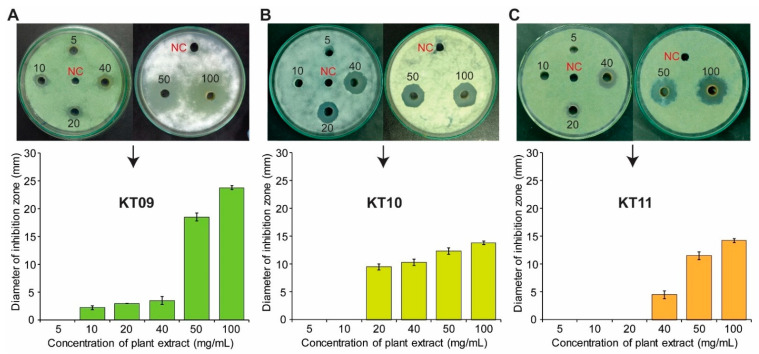
Evaluating the antifungal efficacy of the three selected crude extracts from *M. bealei* (KT09) (**A**), *F. semicordata* (KT10) (**B**), and *G. montanum* (KT11) (**C**) against *P. digitatum* using the agar well diffusion method. The plant extracts were prepared at concentrations of 5–100 mg/mL. A 50 µL volume of fungal spore suspension (10^6^ spores/mL) was spread onto a PDA plate, and 6-mm-diameter wells were punched and filled with 50 µL of each extract. DMSO (10%) was used as the negative control (NC). The plates were incubated at 25–30 °C for 3–4 days. Experiments were performed in triplicate, and data are presented as means ± standard deviations (SD).

To more precisely evaluate the fungal inhibition of the three plant extracts against *P. digitatum*, these extracts were prepared at concentrations of 5–100 mg/mL and tested for antifungal activity using the agar well diffusion method. The results confirmed that *M. bealei* ethanolic extract at a concentration of 100 mg/mL provided a strong antifungal activity against *P. digitatum*, with a diameter of 23.75 ± 0.35 mm. At the same concentrations of 100 mg/mL, both the water extract from *F. semicordata* and the ethanolic extract from *G. montanum* exhibited significant activity against *P. digitatum*, with an inhibition diameter of approximately 14 mm. Notably, the *M. bealei* crude extract showed the antagonistic ability at a lower concentration of 10 mg/mL, compared to the other crude extracts, which required a two-fold higher concentration of 20 mg/mL. At the concentration of 5 mg/mL, no antifungal activity was detected for any of the three extracts (Figure 1).

**Figure 2:**
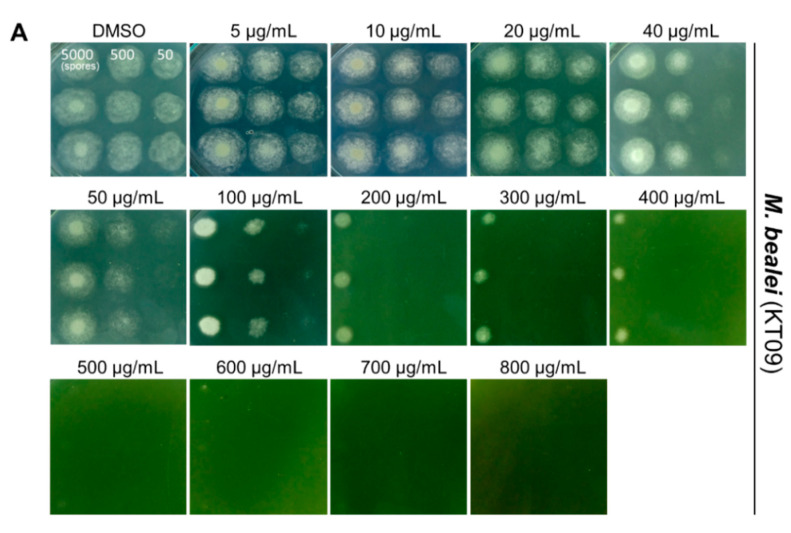

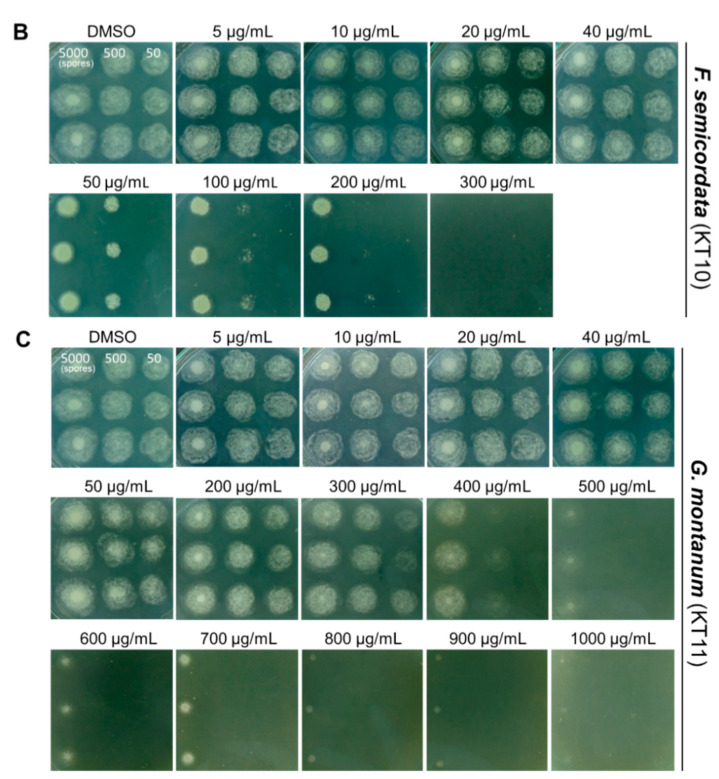
Assays for minimal inhibitory concentrations of the selected crude extracts, including (**A**) *M. bealei* (KT09), (**B**) *F. semicordata* (KT10), and (**C**) *G. montanum* (KT11) against *P. digitatum* using the microdilution method. The crude extracts were added to the PDA medium to obtain final concentrations of 5–1000 µg/mL. DMSO was used as the negative control. Fungal spores were prepared at concentrations of 10^4^, 10^5^, and 10^6^ spores/mL. Then, 5 µL volumes of the spore suspensions (equal to 50, 500, and 5000 spores) were spotted onto the PDA plates. Plates were incubated at 25 °C for 3 days.

To determine minimal inhibitory concentrations (MICs), the extracts were added to the PDA medium to obtain final concentrations of 5–800 µg/mL, and the growth of different inoculums of *P. digitatum* was observed. The results of the microdilution assays indicate that the growth of *P. digitatum* was inhibited by all three plant extracts in a concentration-dependent manner. The KT09 extract displayed no antifungal activity at 5–20 µg/mL; however, at 40–100 µg/mL, the growth of *P. digitatum* with the inoculum of 50 spores was strongly repressed. At 200–400 µg/mL, the fungal growth with inoculums of 50–500 spores was completely inhibited. Further examinations revealed that the pathogen *P. digitatum* hardly grew with the inoculation of 5000 spores on the medium containing the KT09 concentrations of 500–700 µg/mL. No fungal growth could be detected for any of the tested spore inoculums at an extract concentration of 800 µg/mL (Figure 2A). 

Although the water extract of *F. semicordata* (KT10) did not show any inhibitory activity against *P. digitatum* at 5–40 µg/mL, this plant extract strongly inhibited the growth of the pathogen at 50–200 µg/mL. With supplementation of the KT10 aqueous extract at a concentration 300 µg/mL, the proliferation of *P. digitatum* was blocked entirely, even with an inoculum of 5000 spores (Figure 2B). The ethanolic extract of *G. montanum* (KT11) exhibited significant antagonistic activity towards the hyphal extension of *P. digitatum* at concentrations of over 400 µg/mL. With supplementation of KT11 ethanolic extract at 1000 µg/mL, the fungal growth from an inoculum of 5000 spores was almost completely inhibited (Figure 2C).

### 3.3. Three Selected Plant Extracts Suppress the Invasion of P. digitatum on Citrus Fruits

**Figure 3:**
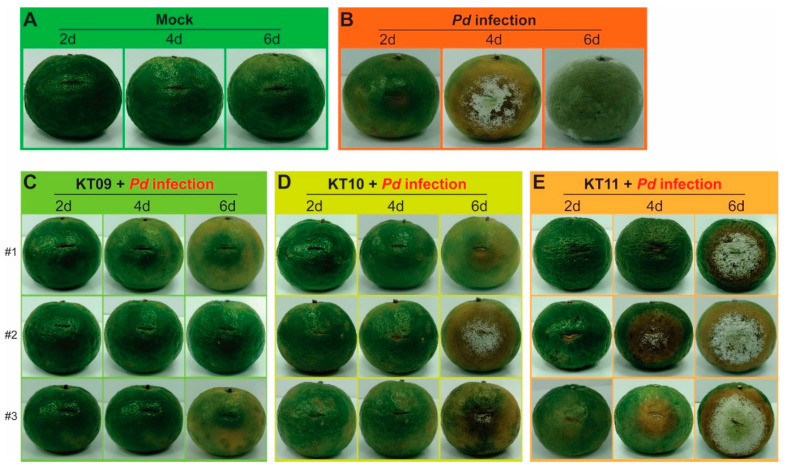
Effect of three selected plant extracts against the postharvest pathogen *P. digitatum* (*Pd*) on orange fruits. The wounded oranges treated with sterile water were used as negative, mock-infected control (**A**), and infected but untreated fruits acted as the positive control (**B**). The wounded fruits were soaked in a solution containing 800 µg/mL of the KT09 ethanolic extract (**C**), 300 µg/mL of the KT10 water extract (**D**), or 1000 µg/mL of the KT11 ethanolic extract (**E**) before infection with *P. digitatum* spores. Experiments were conducted in triplicate. The treated fruits were incubated in sterile plastic boxes at 25 °C for 2–6 days. Three different fruits (#1, #2, and #3) were used as examples for each extract treatment.

The plant extracts from *M. bealei*, *F. semicordata*, and *G. montanum* (KT09, KT10, KT11) were tested for their ability to directly suppress the postharvest pathogen *P. digitatum* on orange fruits. Oranges were wounded and treated separately with the extracts at their respective MICs prior to being inoculated with *P. digitatum* spores. Wounded oranges were treated with sterile water to serve as controls. In comparison to the mock-infected controls (mock) (Figure 3A), the infected oranges were strongly infected with *P. digitatum* at 4 days post infection (dpi), and the fruits were macerated and fully covered with fungal mycelium at 6 dpi (Figure 3B). In contrast, oranges treated with KT09 extract were protected from the invasion of *P. digitatum* (Figure 3C). The wounded citrus fruits soaked in KT10 extract were also wholly protected at 4 dpi and partially at 6 dpi (Figure 3D). However, the citrus fruit protective efficacy of KT11 extract against *P. digitatum* was only mild when compared to the KT09 and KT10 extracts (Figure 3E).

### 3.4. Berberine of the M. bealei Ethanolic Extract as a Major Antifungal Compound

**Figure 4:**
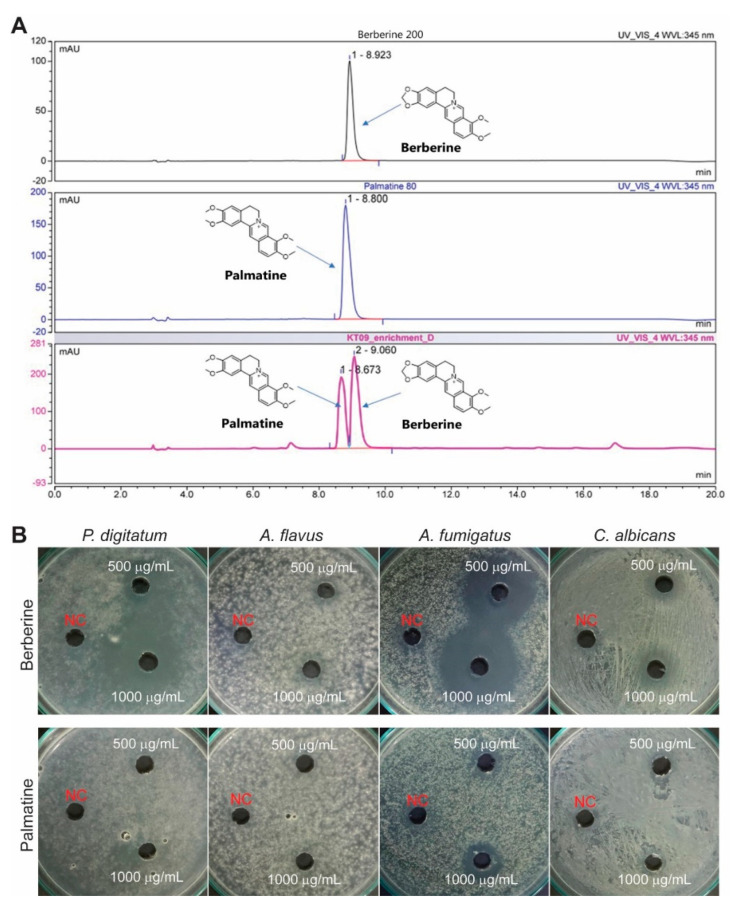
Analysis of the major metabolites of the *M. bealei* ethanolic extract. (**A**) Absorption peaks of berberine and palmatine were detected at 345 nm by HPLC using the standard compounds and alkaloid enrichment. (**B**) Evaluation of the antifungal activity of berberine and palmatine purified from the enrichment extract. These compounds were examined for their antagonistic ability against four different fungi using the agar well diffusion method. Purified berberine and palmatine at concentrations of 500–1000 µg/mL were used for the tests. DMSO (10%) was used for the negative control (NC). Plates were incubated at 25–30 °C for 1–3 days.

The medicinal plant *M. bealei* has been reported to contain high contents of alkaloids, in which berberine and palmatine are the major active compounds [44,45]. Quantifications by HPLC in comparison to the standard compounds (Figure 4A) indicate that the ethanolic crude extract corresponded to 121.2 ± 8.7 mg/g for berberine (approximately 12% of the extract) and 15.2 ± 0.2 mg/g for palmatine (approximately 1.5% of the crude extract). From 20.5 g of the *M. bealei* ethanolic extract, we obtained about 8.6 g of alkaloid enrichment. The contents of berberine and palmatine in the enrichment were significantly higher than those in the extract, with 332.1 ± 1.83 mg/g and 38.67 ± 0.37 mg/g, respectively. Berberine and palmatine were isolated from the alkaloid enrichment using column chromatography methods and were identified using HPLC-DAD analysis under the same conditions as those used for the reference compounds in our in-house library. After purification via the HPLC method, the isolated compounds berberine and palmatine reached a purity of over 97.5%.

Further experiments were performed to determine the antifungal activity of the purified berberine and palmatine. At concentrations of 500–1000 µg/mL, berberine exhibited strong antagonistic activity against the plant pathogen *P. digitatum* and human pathogen *A. fumigatus*, and mild antifungal activity toward *A. flavus* and *C. albicans* (Figure 4B). We also found that at 500–1000 µg/mL, palmatine displayed mild antifungal activity toward *A. fumigatus* (Figure 4B). On the basis of these assays, our data support the finding that berberine is a key compound responsible for the antifungal activity of the *M. bealei* extract.

## 4. Discussion

Medicinal plants have made significant contributions to disease treatment in both traditional and modern healthcare systems. Many bioactive compounds from medicinal plants have been discovered and purified to produce drugs, such as artemisinin from *Artemisia annua* for antimalarial activity, paclitaxel from *Taxus brevifolia* for antitumor activity, vinblastine from *Catharanthus roseus* for anticancer chemotherapy, and berberine from different plant genera for antimicrobial activity [46]. Due to safety and ecological friendliness, plant extracts and their bioactive metabolites are emerging as potential alternatives to synthetic fungicides for controlling fungal pathogens [21,26,27,47]. In the present study, we inspected twelve medicinal plants in Northern Vietnam for their antagonistic behavior toward four common pathogenic fungi causing diseases in humans and plants, including *P. digitatum*, *A. flavus*, *A. fumigatus*, and *C. albicans*. We found that the ethanolic crude extract of *M. bealei* was able to inhibit the growth of all of the tested fungi, but with stronger activity toward *P. digitatum* and *A. fumigatus*. Further results indicated that the ethanolic extract from another plant, *G. montanum*, possessed inhibitory activity against the filamentous fungal pathogens, except for the yeast *C. albicans*. The *G. montanum* extract also displayed vigorous activity against *P. digitatum*. Exceptionally, only *P. digitatum* was able to sense the antifungal activity of the *F. semicordata* water extract, while the other tested fungi could not do that (Table 2, Figure 1). Our data suggest that the citrus postharvest pathogen *P. digitatum* can be employed as a model fungus for detecting the antifungal activity of plant extracts.

Plant extracts have been widely reported for antifungal activities against pathogenic fungi from different geographical regions [11,12,13,14,15]. However, this is the first report on three Vietnamese medicinal plants possessing antifungal ability. So far, there is no information on the antifungal activities of *F. semicordata* and *G. montanum*. *Ficus* species are exploited for dietary uses and medicinal purposes. Crude extracts from *Ficus* plants are frequently used to treat diarrhea, stomach diseases, and diabetes. However, antibacterial and antifungal activities have been reported for only three *Ficus* species, *F. sur*, *F. hirta* and *F. thonningii* [48,49]. Meanwhile, the genus *Gnetum* is well known for polyphenols, and *G. montanum* has traditionally been used to treat arthritis and bronchitis. Numerous constituents of *G. montanum* have been identified, and alkaloids from this plant have been confirmed with respect to their antibacterial activity against *Pseudomonas aeruginosa* and *Staphylococcus aureus* [50,51]. Our present study revealed for the first time that the water extract of *F. semicordata* and the ethanolic extract of *G. montanum* could inhibit *P. digitatum* (Table 2, Figure 1). The microdilution assays indicated that the MIC of the *F. semicordata* water extract for the complete inhibition of *P. digitatum* was relatively low, with only 300 µg/mL when compared to the MICs (800–1000 µg/mL) of the ethanolic extracts from *M. bealei* and *G. montanum* (Figure 2). However, potential metabolites responsible for the antifungal activities of these extracts still need to be clarified in the future.

Several plant extracts and their metabolites have been demonstrated to be effective as natural preservatives for fruits against pathogenic *Penicillium* species [9,52]. We used the MIC values to examine the abilities of the crude extracts from *M. bealei*, *F. semicordata*, and *G. montanum* in the preservation of orange fruits. The results reveal that these plant extracts are able to protect the fruits from infection with *P. digitatum* to different degrees, whereby the extracts from *M. bealei* and *F. semicordata* exhibited higher protective efficacies than the extract from *G. montanum* (Figure 3). Among the selected extracts, the *M. bealei* ethanolic extract with broad-spectrum antifungal activity was further analyzed to identify potential compounds responsible for the fungal inhibitory effects. *M. bealei*, an evergreen shrub of the Berberidaceae family, native to East Asia and North and Central America, is cultivated in Northern Vietnam. This plant has been used in traditional medicine for a long time, as it contains benzylisoquinoline alkaloids, including berberine, berbamin, oxyacanthin, isotetradrin, palmatine, and jatrorrhizine. Several publications have reported various pharmacological properties of these alkaloids, including antibacterial, anticancer, and anti-inflammatory activities [44,53,54,55]. In the current study, berberine and palmatine were isolated from the ethanolic extract of *M. bealei* and evaluated for their antifungal activities. According to the quantitative results, berberine appeared to be a major constituent, accounting for about 12% of the *M. bealei* ethanolic extract. Berberine showed similar antimicrobial activity to that of *M. bealei* ethanolic extract, while palmatine had mild antifungal activity at the inspected doses (Figure 4). Berberine can be extracted from the roots, stem barks, and branches of various *Berberis* species, as well as from other medicinal plants [16,44,56]. This active compound is considered very effective in pharmacology, and the abundance of plant sources for this compound has been investigated. Therefore, it is likely to find more applications in other areas, particularly agriculture, as a potential agent for the postharvest preservation of fruits.

## 5. Conclusions

In this study, twelve medicinal plants collected in Northern Vietnam were screened for antifungal activities against four different pathogenic fungi, including *C. albicans*, *A. fumigatus*, *A. flavus*, and *P. digitatum*. In particular, the antifungal activities of the extracts from *M. bealei*, *F. semicordata*, and *G. montanum* could easily be detected for *P. digitatum*. These crude extracts also represent potential preservatives for protecting citrus fruits from the invasion of the postharvest pathogen *P. digitatum*. Furthermore, we found that the *M. bealei* ethanolic extract possessed strong antifungal activity against all four of the tested fungi. Chemical analyses of this extract by HPLC revealed that it contained berberine and palmatine as major metabolites, and berberine appeared to be the key compound for the antifungal activity of the extract.

## Figures and Tables

**Table 1:** Medicinal plants used in this study.

Plant Species (Family)	Plant Sample Codes	Parts for Extraction	Period of Sample Collection	Location of Sample Collection	Specimen Collection Codes
*Clerodendrum cyrtophyllum* Turcz. (Verbenaceae)	KT07	Leaf	December 2017	Ha Giang	HNU 024106
*Mahonia bealei* (Fortune) Carrière (Berberidaceae)	KT09	Stem	October 2017	Lao Cai	HNU 024779
*Ficus semicordata* Buch.-Ham. ex Sm. (Moraceae)	KT10	Leaf, stem	May 2017	Ha Giang	HNU 024780
*Gnetum montanum* Markgr. (Gnetaceae)	KT11	Stem	May 2017	Ha Giang	HNU 024781
*Tacca chantrieri* André (Dioscoreaceae)	KT12	Root	May 2017	Ha Giang	HNU 024782
*Crinum asiaticum* L. (Amaryllidaceae)	KT13	Leaf	June 2017	Quang Ninh	HNU 024783
*Mallotus barbatus* Müll.Arg. (Euphorbiaceae)	KT14	Root	June 2017	Hoa Binh	HNU 024784
*Aganope balansae* (Gagnep.) P.K.Lôc (Fabaceae)	KT15	Stem	October 2018	Ha Giang	HNU 024785
*Hedyotis capitellata* Wall. ex G.Don (Rubiaceae)	KT16	Leaf, stem	July 2018	Ha Giang	HNU 024786
*Stixis scandens* Lour. (Capparaceae)	KT17	Leaf	August 2019	Ha Giang	HNU 024787
*Cymbidium aloifolium* (L.) Sw. (Orchidaceae)	KT18	Leaf, stem	August 2018	Tuyen Quang	HNU 024788
*Tinospora sinensis* (Lour.) Merr. (Menispermaceae)	KT20	Stem	November 2018	Hoa Binh	HNU 024790

**Table 2:** Antifungal activity of the plant crude extracts.

Plant Species	Plant Sample Codes	Water Extract				Ethanolic Extract			
		*Pd*	*Afl*	*Afu*	*Ca*	*Pd*	*Afl*	*Afu*	*Ca*
*C. cyrtophyllum*	KT07	−	−	−	−	−	−	−	−
*M. bealei*	KT09	−	−	−	−	+++	+	+++	++
*F. semicordata*	KT10	++	−	−	−	−	−	−	−
*G. montanum*	KT11	−	−	−	−	++	+	+	−
*T. chantrieri*	KT12	−	−	−	−	−	−	−	−
*C. asiaticum*	KT13	−	−	−	−	−	−	−	−
*M. barbatus*	KT14	−	−	−	−	−	−	−	−
*A. balansae*	KT15	−	−	−	−	−	−	−	−
*H. capitellata*	KT16	−	−	−	−	−	−	−	−
*S. scandens*	KT17	−	−	−	−	−	−	−	−
*C. aloifolium*	KT18	−	−	−	−	−	−	−	−
*T. sinensis*	KT20	−	−	−	−	−	−	−	−

Note: *Pd* (*P. digitatum*), *Afl* (*A. flavus*), *Afu* (*A. fumigatus*), *Ca* (*C. albicans*), (−) no activity, (**+**) mild activity, (**++**) moderate activity, (**+++**) strong activity.

## Data Availability

Not applicable.

## Supplementary Materials

The following supporting information can be downloaded at: https://www.mdpi.com/article/10.3390/jof8090956/s1, Figure S1: Medicinal plants were collected in Northern Vietnam to examine antifungal activity, Figure S2: Antifungal activity of the *M. bealei* (KT09) ethanolic extract against other pathogenic fungi.

## References

[1] Kurnaz M.L., Kurnaz I.A. (2021). Commercialization of medicinal bioeconomy resources and sustainability. Sustain. Chem. Pharm..

[2] Astutik S., Pretzsch J., Kimengsi J.N. (2019). Asian medicinal plants’ production and utilization potentials: A review. Sustainability.

[3] Patra J.K., Das G., Lee S., Kang S.S., Shin H.S. (2018). Selected commercial plants: A review of extraction and isolation of bioactive compounds and their pharmacological market value. Trends Food Sci. Technol..

[4] Azmir J., Zaidul I.S.M., Rahman M.M., Sharif K.M., Mohamed A., Sahena F., Jahurul M.H.A., Ghafoor K., Norulaini N.A.N., Omar A.K.M. (2013). Techniques for extraction of bioactive compounds from plant materials: A review. J. Food Eng..

[5] Yusoff I.M., Taher Z.M., Rahmat Z., Chua L.S. (2022). A review of ultrasound-assisted extraction for plant bioactive compounds: Phenolics, flavonoids, thymols, saponins and proteins. Food Res. Int..

[6] Yang L., Yang C., Li C., Zhao Q., Liu L., Fang X., Chen X.Y. (2016). Recent advances in biosynthesis of bioactive compounds in traditional Chinese medicinal plants. Sci. Bull..

[7] Porras G., Chassagne F., Lyles J.T., Marquez L., Dettweiler M., Salam A.M., Samarakoon T., Shabih S., Farrokhi D.R., Quave C.L. (2020). Ethnobotany and the role of plant natural products in antibiotic drug discovery. Chem. Rev..

[8] Geddes-McAlister J., Shapiro R.S. (2019). New pathogens, new tricks: Emerging, drug-resistant fungal pathogens and future prospects for antifungal therapeutics. Ann. N. Y. Acad. Sci..

[9] Bhatta U.K. (2022). Alternative management approaches of citrus diseases caused by *Penicillium digitatum* (green mold) and *Penicillium italicum* (blue mold). Front. Plant Sci..

[10] Fisher M.C., Alastruey-Izquierdo A., Berman J., Bicanic T., Bignell E.M., Bowyer P., Bromley M., Brüggemann R., Garber G., Cornely O.A. (2022). Tackling the emerging threat of antifungal resistance to human health. Nat. Rev. Microbiol..

[11] Ali-Shtayeh M.S., Yaghmour R.M.R., Faidi Y.R., Salem K., Al-Nuri M.A. (1998). Antimicrobial activity of 20 plants used in folkloric medicine in the Palestinian area. J. Ethnopharmacol..

[12] Giordani C., Simonetti G., Natsagdorj D., Choijamts G., Ghirga F., Calcaterra A., Quaglio D., De Angelis G., Toniolo C., Pasqua G. (2019). Antifungal activity of Mongolian medicinal plant extracts. Nat. Prod. Res..

[13] Webster D., Taschereau P., Belland R.J., Sand C., Rennie R.P. (2008). Antifungal activity of medicinal plant extracts; preliminary screening studies. J. Ethnopharmacol..

[14] Hoffman B.R., DelasAlas H., Blanco K., Wiederhold N., Lewis R.E., Williams L. (2008). Screening of antibacterial and antifungal activities of ten medicinal plants from Ghana. Pharm. Biol..

[15] Dabur R., Singh H., Chhillar A.K., Ali M., Sharma G.L. (2004). Antifungal potential of Indian medicinal plants. Fitoterapia.

[16] Imanshahidi M., Hosseinzadeh H. (2008). Pharmacological and therapeutic effects of *Berberis vulgaris* and its active constituent, berberine. Phytother. Res..

[17] Tillhon M., Ortiz L.G., Lombardi P., Scovassi A.I. (2012). Berberine: New perspectives for old remedies. Biochem. Pharmacol..

[18] Aldholmi M., Marchand P., Ourliac-Garnier I., Le Pape P., Ganesan A. (2019). A decade of antifungal leads from natural products: 2010–2019. Pharmaceuticals.

[19] Corkley I., Fraaije B., Hawkins N. (2021). Fungicide resistance management: Maximizing the effective life of plant protection products. Plant Pathol..

[20] Khetabi A.E., Lahlali R., Ezrari S., Radouane N., Lyousfi N., Banani H., Askarne L., Tahiri A., Ghadraoui L.E., Belmalha S. (2022). Role of plant extracts and essential oils in fighting against postharvest fruit pathogens and extending fruit shelf life: A review. Trends Food Sci. Technol..

[21] Makhuvele R., Naidu K., Gbashi S., Thipe V.C., Adebo O.A., Njobeh P.B. (2020). The use of plant extracts and their phytochemicals for control of toxigenic fungi and mycotoxins. Heliyon.

[22] Wu Y., Ren D., Gao C., Li J., Du B., Wang Z., Qian S. (2021). Recent advances for alkaloids as botanical pesticides for use in organic agriculture. Int. J. Pest Manag..

[23] Seepe H.A., Nxumalo W., Amoo S.O. (2021). Natural products from medicinal plants against phytopathogenic *Fusarium* species: Current research endeavours, challenges and prospects. Molecules.

[24] Costa J.H., Bazioli J.M., de Moraes Pontes J.G., Fill T.P. (2019). *Penicillium digitatum* infection mechanisms in citrus: What do we know so far?. Fungal Biol..

[25] Sánchez-Torres P. (2021). Molecular mechanisms underlying fungicide resistance in citrus postharvest green mold. J. Fungi.

[26] Matrose N.A., Obikeze K., Belay Z.A., Caleb O.J. (2021). Plant extracts and other natural compounds as alternatives for post-harvest management of fruit fungal pathogens: A review. Food Biosci..

[27] Ngegba P.M., Cui G., Khalid M.Z., Zhong G. (2022). Use of botanical pesticides in agriculture as an alternative to synthetic pesticides. Agriculture.

[28] Do T.L., Nguyen X.D. (1991). Native drugs of Vietnam: Which traditional and scientific approaches?. J. Ethnopharmacol..

[29] Hoang S.V., Baas P., Keßler P.J.A. (2008). Traditional medicinal plants in Ben En National Park, Vietnam. Blumea.

[30] On T.V., Quyen D., Bich L.D., Jones B., Wunder J., Russell-Smith J. (2001). A survey of medicinal plants in BaVi National Park, Vietnam: Methodology and implications for conservation and sustainable use. Biol. Conserv..

[31] Giang P.M., Otsuka H. (2018). New compounds and potential candidates for drug discovery from medicinal plants of Vietnam. Chem. Pharm. Bull..

[32] Lee C., Kim S.Y., Eum S., Paik J.H., Bach T.T., Darshetkar A.M., Choudhary R.K., Hai D.V., Quang B.H., Thanh N.T. (2019). Ethnobotanical study on medicinal plants used by local Van Kieu ethnic people of Bac Huong Hoa nature reserve, Vietnam. J. Ethnopharmacol..

[33] Nguyen X.M.A., Bun S.S., Ollivier E., Dang T.P.T. (2020). Ethnobotanical study of medicinal plants used by K’Ho-Cil people for treatment of diarrhea in Lam Dong Province, Vietnam. J. Herb. Med..

[34] Banskota A.H., Tezuka Y., Le Tran Q., Kadota S. (2003). Chemical constituents and biological activities of Vietnamese medicinal plants. Curr. Top. Med. Chem..

[35] Ueda J.Y., Tezuka Y., Banskota A.H., Tran Q.L., Tran Q.K., Harimaya Y., Saiki I., Kadota S. (2002). Antiproliferative activity of Vietnamese medicinal plants. Biol. Pharm. Bull..

[36] Nguyen M.T.T., Awale S., Tezuka Y., Tran Q.L., Watanabe H., Kadota S. (2004). Xanthine oxidase inhibitory activity of Vietnamese medicinal plants. Biol. Pharm. Bull..

[37] Nguyen-Pouplin J., Tran H., Tran H., Phan T.A., Dolecek C., Farrar J., Tran T.H., Caron P., Bodo B., Grellier P. (2007). Antimalarial and cytotoxic activities of ethnopharmacologically selected medicinal plants from South Vietnam. J. Ethnopharmacol..

[38] Nguyen Q.V., Nguyen V.B., Eun J.B., Wang S.L., Nguyen D.H., Tran T.N., Nguyen A.D. (2016). Anti-oxidant and antidiabetic effect of some medicinal plants belong to *Terminalia* species collected in Dak Lak Province, Vietnam. Res. Chem. Intermed..

[39] Vu T.T., Kim H., Tran V.K., Dang Q.L., Nguyen H.T., Kim H., Kim I.S., Choi G.J., Kim J.C. (2016). In vitro antibacterial activity of selected medicinal plants traditionally used in Vietnam against human pathogenic bacteria. BMC Complementary Altern. Med..

[40] Nguyen-Vo T.H., Le T., Pham D., Nguyen T., Le P., Nguyen A., Nguyen T., Nguyen T.N., Nguyen V., Do H. (2018). VIETHERB: A database for Vietnamese herbal species. J. Chem. Inf. Modeling.

[41] Vu X.T., Ngo T.T., Mai T.D.L., Bui T.T., Le H.D., Bui T.V.H., Nguyen Q.H., Ngo X.B., Tran V.T. (2018). A highly efficient *Agrobacterium tumefaciens*-mediated transformation system for the postharvest pathogen *Penicillium digitatum* using DsRed and GFP to visualize citrus host colonization. J. Microbiol. Methods.

[42] Nierman W.C., Yu J., Fedorova-Abrams N.D., Losada L., Cleveland T.E., Bhatnagar D., Bennett J.W., Dean R., Payne G.A. (2015). Genome sequence of *Aspergillus flavus* NRRL 3357, a strain that causes aflatoxin contamination of food and feed. Genome Announc..

[43] Balouiri M., Sadiki M., Ibnsouda S.K. (2016). Methods for in vitro evaluating antimicrobial activity: A review. J. Pharm. Anal..

[44] Huang Y., Wang T., Jiang Z. (2021). Fast analysis of alkaloids from different parts of *Mahonia bealei* (Fort.) Carr. studied for their anti-Alzheimer’s activity using supercritical fluid chromatography. J. Sep. Sci..

[45] He J.M., Mu Q. (2015). The medicinal uses of the genus *Mahonia* in traditional Chinese medicine: An ethnopharmacological, phytochemical and pharmacological review. J. Ethnopharmacol..

[46] Gurib-Fakim A. (2006). Medicinal plants: Traditions of yesterday and drugs of tomorrow. Mol. Asp. Med..

[47] Liu Q., Luyten W., Pellens K., Wang Y., Wang W., Thevissen K., Liang Q., Cammue B.P.A., Schoofs L., Luo G. (2012). Antifungal activity in plants from Chinese traditional and folk medicine. J. Ethnopharmacol..

[48] Shi Y., Mon A.M., Fu Y., Zhang Y., Wang C., Yang X., Wang Y. (2018). The genus *Ficus* (Moraceae) used in diet: Its plant diversity, distribution, traditional uses and ethnopharmacological importance. J. Ethnopharmacol..

[49] Wan C., Chen C., Li M., Yang Y., Chen M., Chen J. (2017). Chemical constituents and antifungal activity of *Ficus hirta* Vahl. fruits. Plants.

[50] Martin F., Grkovic T., Sykes M.L., Shelper T., Avery V.M., Camp D., Quinn R.J., Davis R.A. (2011). Alkaloids from the Chinese Vine *Gnetum montanum*. J. Nat. Prod..

[51] Xiang W., Jiang B., Li X.M., Zhang H.J., Zhao Q.S., Li S.H., Sun H.D. (2002). Constituents of *Gnetum montanum*. Fitoterapia.

[52] Chen J., Shen Y., Chen C., Wan C. (2019). Inhibition of key citrus postharvest fungal strains by plant extracts in vitro and in vivo: A review. Plants.

[53] Hu W., Yu L., Wang M.H. (2011). Antioxidant and antiproliferative properties of water extract from *Mahonia bealei* (Fort.) Carr. leaves. Food Chem. Toxicol..

[54] Hu W., Wu L., Qiang Q., Ji L., Wang X., Luo H., Wu H., Jiang Y., Wang G., Shen T. (2016). The dichloromethane fraction from *Mahonia bealei* (Fort.) Carr. leaves exerts an anti-inflammatory effect both in vitro and in vivo. J. Ethnopharmacol..

[55] Wu L., Shen T., Zhou Y., Wu J., Ji X.Y., Si C.L., Hu W.C. (2018). Secondary metabolites of *Mahonia bealei* branches. Chem. Nat. Compd..

[56] Singh I.P., Mahajan S. (2012). Berberine and its derivatives: A patent review (2009–2012). Expert Opin. Ther. Pat..

