# Supplementary material for: Penicillium digitatum as a Model Fungus for Detecting Antifungal Activity of Botanicals: An Evaluation on Vietnamese Medicinal Plant Extracts
Source: J Fungi (Basel). 2022 Sep 13;8(9):956. doi: 10.3390/jof8090956 (PMC9502062; doi:10.3390/jof8090956)
Supplement: Supplementary file 1 [file jof-08-00956-s001.zip › jof-1871482-supplementary.pdf]

Article

Penicillium digitatum as a Model Fungus for Detecting Antifungal Activity of Botanicals: An Evaluation on Vietnamese Medicinal Plant Extracts

Hanh My Tran [1,2], Diep Hong Le [1], Van-Anh Thi Nguyen [1], Tao Xuan Vu [3], Nguyen Thi Kim Thanh [1], Do Hoang Giang [4], Nguyen Tien Dat [4], Hai The Pham [1], Marc Muller [2,*], Huy Quang Nguyen [1,5,*] and Van-Tuan Tran [1,5,*]

[1] Faculty of Biology, University of Science, Vietnam National University, Hanoi (VNU), 334 Nguyen Trai, Thanh Xuan, Hanoi 100000, Vietnam
[2] Laboratory for Organogenesis and Regeneration, Department of Life Sciences, GIGA-I3, University of Liège, 4000 Liège, Belgium
[3] Center for Experimental Biology, National Center for Technological Progress, Ministry of Science and Technology, C6 Thanh Xuan Bac, Thanh Xuan, Hanoi 100000, Vietnam
[4] Center for Research and Technology Transfer, Vietnam Academy of Science and Technology, 18 Hoang Quoc Viet, Cau Giay, Hanoi 100000, Vietnam
[5] National Key Laboratory of Enzyme and Protein Technology, University of Science, Vietnam National University, Hanoi (VNU), 334 Nguyen Trai, Thanh Xuan, Hanoi 100000, Vietnam
* Correspondence: tuantran@vnu.edu.vn (V.-T.T.); m.muller@uliege.be (M.M.); nguyenquanghuy@vnu.edu.vn (H.Q.N.)

SUPPLEMENTARY MATERIALS

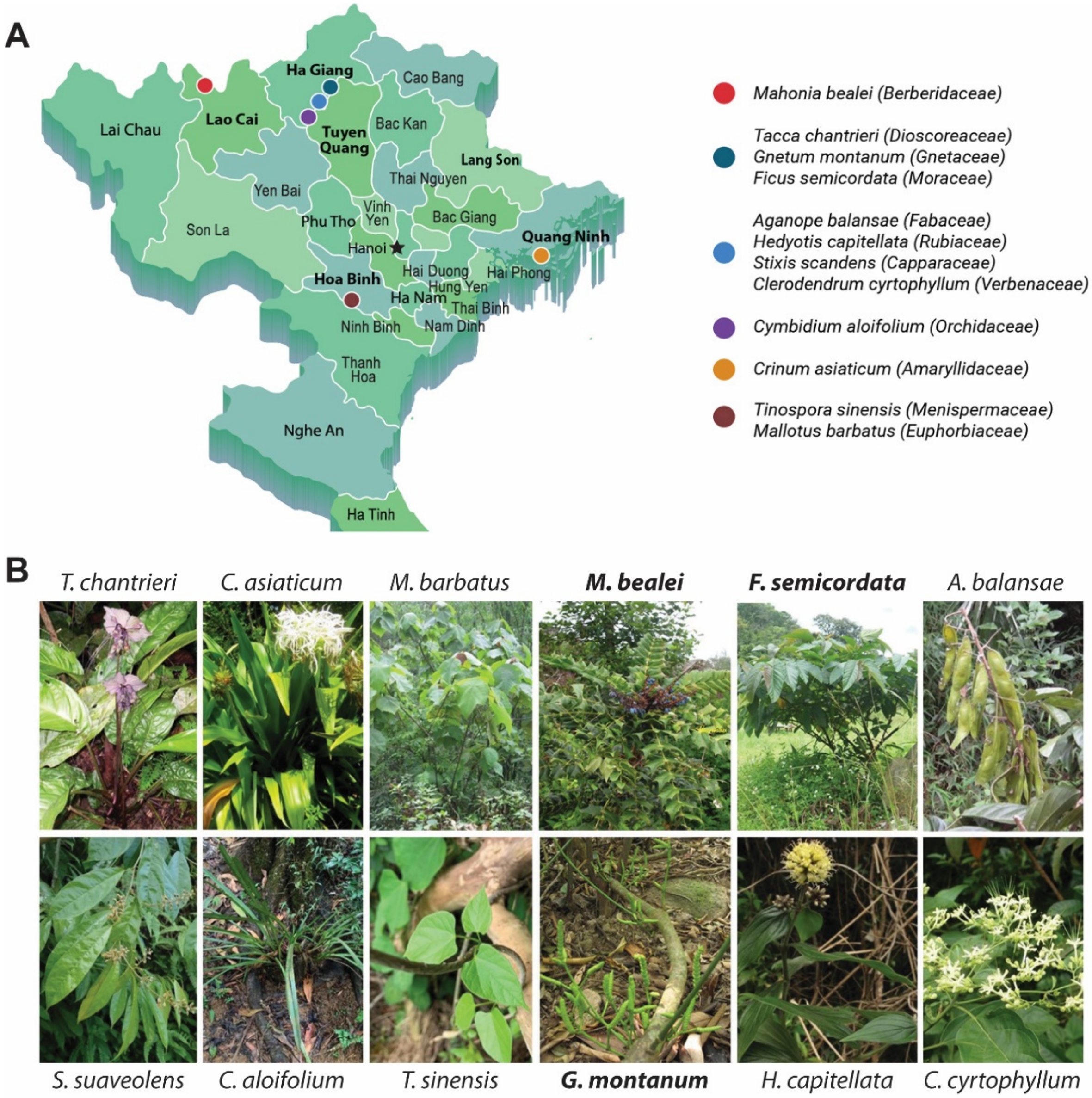

Figure S1. Medicinal plants were collected in Northern Vietnam to examine antifungal activity. (**A**) Geographical locations of twelve plants in five different provinces of Northern Vietnam, including Lao Cai, Ha Giang, Quang Ninh, Hoa Binh, and Tuyen Quang. (**B**) The representative morphologies of the twelve medicinal plant species.

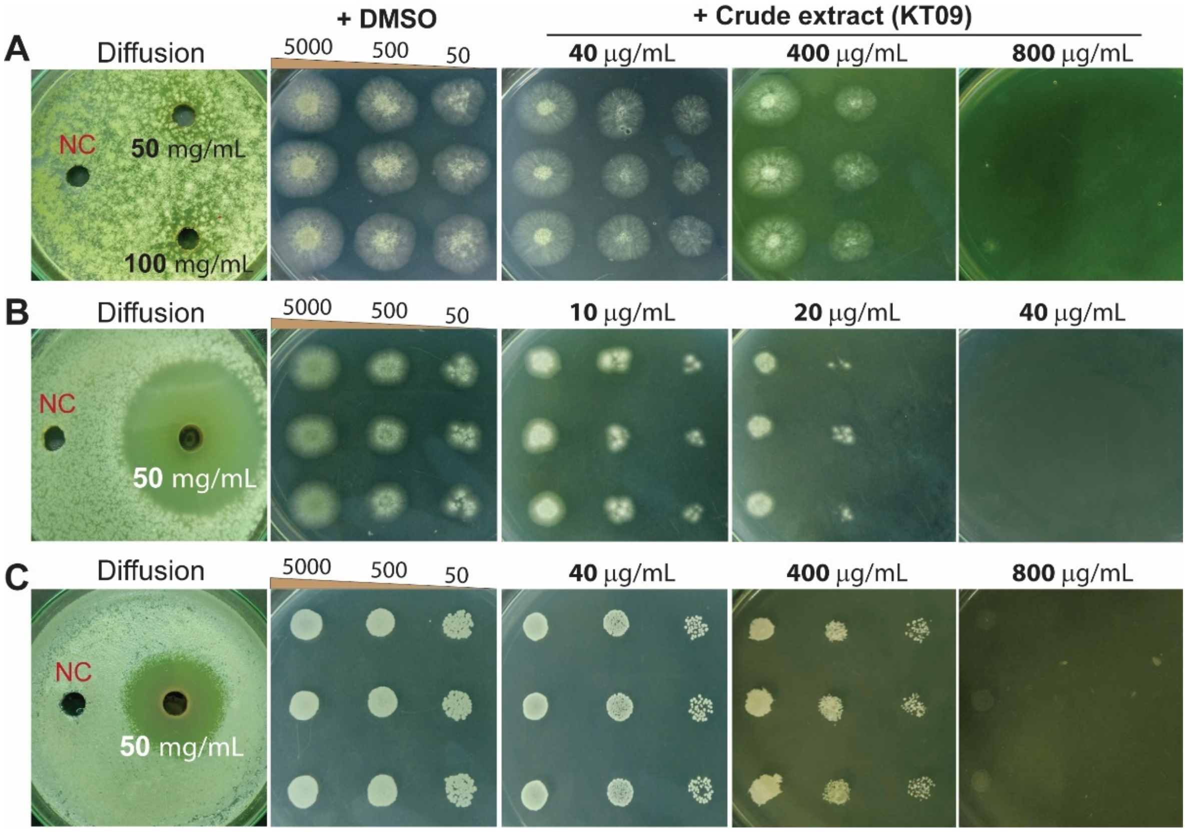

Figure S2. Antifungal activity of the *M. bealei* (KT09) ethanolic extract against other pathogenic fungi, including (**A**) the aflatoxin-producing mold *A. flavus*, (**B**) the human pathogenic filamentous fungus *A. fumigatus*, and (**C**) the human pathogenic yeast *C. albicans*. For the agar plate diffusion method, 50 μL of crude extract (50 mg/mL or 100 mg/mL) was filled in agar well. For the microdilution method, spore suspension volumes corresponding to 50, 500, and 5000 fungal spores or yeast cells were spotted on the PDA medium containing different concentrations of the plant extracts. DMSO (10%) was used for the negative control (NC). Plates were incubated at 30 °C or 37 °C for 1–3 days.
